# Two dimensional gel phosphoproteome of peripheral blood mononuclear cells: comparison between two enrichment methods

**DOI:** 10.1186/s12953-014-0046-1

**Published:** 2014-09-09

**Authors:** Maria Teresa Rocchetti, Michela Alfarano, Leonarda Varraso, Salvatore Di Paolo, Massimo Papale, Elena Ranieri, Giuseppe Grandaliano, Loreto Gesualdo

**Affiliations:** Department of Emergency and Organ Transplantation (DETO), Nephrology, Dialysis and Transplantation Unit, University of Bari Aldo Moro, Piazza G. Cesare, 11, Bari, 70124 Italy; Department of Medical and Surgical Sciences, Proteomics and Mass Spectrometry Core Facility, University of Foggia, Foggia, Italy; Department of Medical and Surgical Sciences, Nephrology, Dialysis and Transplantation Unit, University of Foggia, Foggia, Italy; Nephrology and Dialysis Unit, Hospital Dimiccoli, Barletta, Italy; Clinical Pathology Unit, Department of Medical and Surgical Sciences, University of Foggia, Foggia, Italy

**Keywords:** 2DE, Lanthanum, PBMCs, Phosphoproteome, Phosphoprotein enrichment

## Abstract

**Background:**

Protein phosphorylation is considered a key event in signal transduction. Peripheral blood mononuclear cells (PBMCs) are a critical component of the immune system. The analysis of PBMCs phosphoproteome might help elucidate the signaling pathways essential to their biological role in health, immunological diseases and cancer. Enrichment of phosphoproteins becomes a prerequisite for phosphoproteome analysis and conventionally requires a multi-step procedure and sophisticated equipments. In this study, we standardized 2D-PAGE phosphoproteome analysis of PBMCs and compared two phosphoprotein enrichment methods, lanthanum chloride precipitation and affinity micro-column. Further, the different specificity for PBMCs phosphorylated proteins of each method was investigated.

**Results:**

PBMCs were isolated from fresh whole blood of ten healthy donors. PBMCs phosphoproteins were enriched either by phosphoprotein precipitation using lanthanum chloride, with an overall yield of 8.9 ± 4.7%, or by using an affinity micro-column, with a lower yield of 3.2 ± 1.6% (p = 0.05). Image analysis of Sypro-stained analytical 2D-PAGE gels detected 554 ± 68 protein spots for the lanthanum pattern [inter-assay coefficient of variation (CV) = 27.0%, intra-assay CV = 10.7%] and 575 ± 35 protein spots for the micro-column pattern (inter-assay CV = 26.8%; intra-assay CV = 11.0%) (p = 0.6), with 57% match of the spots detected by the 2 approaches. 1D gel electrophoresis and western blot analyses of the supernatants suggested a better lanthanum ions capability to deplete phosphoproteins in a PBMCs protein lysate compared to the affinity micro-column. On the other hand, 1D gel electrophoresis analysis of dephosphorylated PBMCs protein lysate revealed a relatively higher unspecificity for the lanthanum ions compared to affinity micro-column. Filamin-A, coronin 1A, pyruvate kinase isozymes M1/M2 and ficolin-1 were considerably more expressed in the lanthanum phosphoprotein pattern. Interestingly, ficolin-1 had not been reported in 2DE-PBMCs proteome profiles so far.

**Conclusion:**

Our results describe the differences and the validity of phosphoprotein enrichment methods and provide two successful and complementary approaches for the 2DE phosphoproteome analysis of PBMCs.

**Electronic supplementary material:**

The online version of this article (doi:10.1186/s12953-014-0046-1) contains supplementary material, which is available to authorized users.

## Introduction

Protein phosphorylation represents the main post-translational modification, which modulates the functional activity of proteins in most cellular processes. Around 30-50% proteins are estimated to be reversibly phosphorylated at some point during their lifespan, but only 1% of cellular proteins are phosphorylated at any given time [1]. Given the role of phosphorylation processes in the regulation of most protein activities and properties, its derangement is expected to contribute to the genesis and progression of diseases and, conversely, most of phosphorylated proteins may be potential targets for drug therapy of diseases.

Phosphoproteome analysis is a challenging task essentially because of the low stoichiometry of phosphorylation and the low abundance of phosphoproteins within cells at any given time. Peripheral blood mononuclear cells (PBMCs) are a critical component of the immune system and include lymphocytes, monocytes and dendritic cells. Defining the 2D-map of the phosphoproteins and their isoforms offers an exceptional tool for protein cataloguing, which could reveal the signaling pathways essential to maintain the molecular programs of PBMCs useful for the study of all diseases in which the immunological system is activated [2].

In recent years, gel-based phosphoproteomic analysis used a phospho-specific fluorescent dye which detected phosphoproteins in the presence of the entire protein content [3]. The main limitation of this analytical strategy is the high background of non-phosphorylated proteins, which decreases the sensitivity of phosphoprotein analysis. Therefore, preliminary enrichment of phosphoproteins from complex protein mixtures is highly recommended to reduce sample complexity and thus enhance the accessible dynamic range. Most of the available phosphoprotein enrichment methods focused on chromatographic approaches in which target proteins are separated by metal ions caged in immobilized metal ion affinity chromatographic (IMAC) resin, or by immunoaffinity using antibodies directed against phospho-Serine, phospho-Threonine and phospho-Tyrosine [4]. Both techniques would represent suitable approaches for proteomic studies; however, they present some limitations, such as a rather poor phosphoprotein specificity for IMAC (eluted proteins are > of 30% of the total amount of analyzed proteins) [5], and the high costs for protein purification in a multi milligram scale for the immunoaffinity isolation with anti-phospho aminoacid antibodies.

With this scenario in mind, in the attempt to analyze the phosphoproteome of PBMCs by 2D-PAGE analysis, we enriched the phosphoproteins applying a simple precipitation by lanthanum chloride [6]. Then, the 2DE phosphoproteome map of PBMCs obtained by this approach was compared with the one obtained by PBMCs phosphoproteins isolated by commercially available affinity micro-columns. The 2 enrichment methods display an apparently different chemistry of binding, which would imply qualitative and quantitative differences in phosphoproteins. It is known that lanthanum ions strongly bind the phosphate group. When lanthanum chloride is added to a protein solution, a metal-phosphoprotein chelate forms (and precipitates), in which the negatively charged oxygens of the phospho-residues act as a chelating agent to a metal ion, La^3+^. Conversely, the binding of phosphoproteins to the affinity micro-column takes place onto a solid support, a functionalized resin designed to bind the protein phospho-residues. A description of the validity of the two enrichment methods is also reported.

## Results and discussion

**Figure 1:**
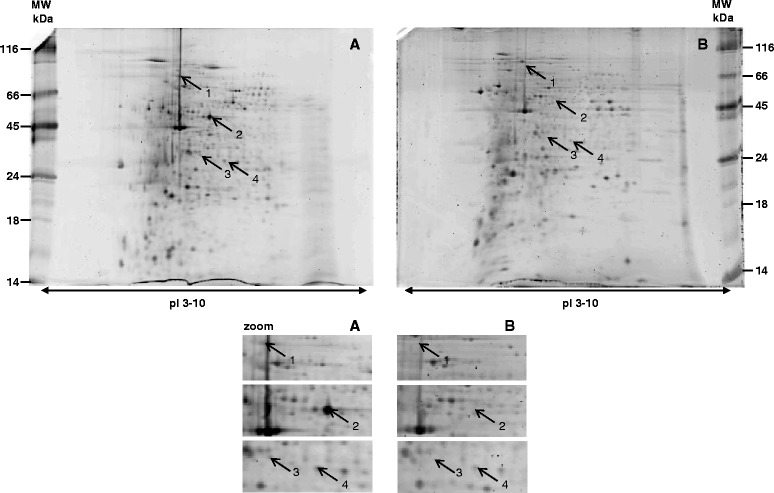
**2DE phosphoproteome of PBMCs.** Representative 2D gel of PBMCs phosphoproteome obtained by isolation of phosphoproteins from total cell lysate using LaCl_3_ **(A)** or affinity micro-columns **(B)**. MW markers:PeppermintStick™ Phosphoprotein MW Standards (Invitrogen™). The arrows indicate four protein spots more expressed in the 2D gel of PBMCs phosphoproteome obtained by isolation of phosphoproteins by LaCl_3_, identified by MALDI-TOF-MS/MS as filamin-A (1), coronin-1A (2), pyruvate kinase isoenzyme M1/M2 (3), and ficolin-1 (4) (Table 1).

PBMCs were isolated from ten healthy individuals with a mean age of 37 ± 11 years and a gender ratio of 5/5 (M/F). Approximately 1.5 (1.56 ± 0.62) mg of total proteins were isolated from each individual. In the first approach, the procedure described by Pink and coworkers [6] was applied and adapted to enrich the phosphoproteome of 4 randomly chosen PBMCs samples. In the second approach, phosphoproteins of 4 different PBMCs samples were enriched by a phosphoprotein enrichment kit of Invitrogen based on a column containing a phosphoprotein-binding resin. Phosphoproteins were isolated from 0.5-1.0 mg of total protein extract with an overall yield of 8.9 ± 4.7% for lanthanum ions and a lower yield of 3.2 ± 1.6% for micro-column (p = 0.05). PBMCs phosphoproteins isolated with each method were then analyzed by 2D-PAGE. Image analysis of the Sypro-stained analytical gels detected 554 ± 68 protein spots for the lanthanum [inter-assay coefficient of variation (CV) = 27.0%, intra-assay CV = 10.7%] and 575 ± 35 protein spots for the micro-column method (inter-assay CV = 26.8%; intra-assay CV = 11.0%) (p = 0.6, Mann Whitney U-test) with almost the same reproducibility (Figure 1). Image analysis revealed that 57% of the detected spots could be matched between the 2DE reference maps obtained by either approach. To underscore the differences between the two isolation strategies, we reported in Table 1 four among all the protein spots identified (data not shown) by MALDI-TOF-MS/MS analysis, chosen on the basis of their highest fold change between the 2 methods, being considerably more expressed in the lanthanum phosphoprotein pattern (Figure 1, Table 1). The 4 proteins spots stained by ProQ Diamond (Additional file 1) were excised from a preparative Coomassie blue stained gel and identified by mass spectrometry as filamin-A, a ubiquitous protein, which anchors various transmembrane proteins to the actin cytoskeleton and serves as a scaffold for a wide range of cytoplasmic signaling proteins [7]; coronin-1A, a cytoplasmic protein involved in T cell homeostasis and actin cytoskeleton organization [8]; pyruvate kinase isoenzyme M1/M2, a kinase involved in carbohydrate degradation [9]; and ficolin-1, a protein expressed on the cell surface of monocytes and granulocytes, which binds activated but not resting T lymphocytes and is a recognition molecule for the lectin complement pathway [10]. Interestingly, ficolin-1 had not been reported in 2DE PBMCs’proteome profiles published so far [11-14], thereby highlighting the usefulness of enrichment approaches for in-depth phosphoprotein analysis.

**Table 1:** **Phosphorylated PBMCs’ proteins differentially expressed between the two phosphoprotein pattern (LaCl**_**3**_ ***versus*** **affinity micro-column)**

**Protein name**	**Accession number**	**Molecular weight (Da)**	**Mascot score**	**Seq. Cov (%)**	**Phosphorylation sites**	**P value** ^**a**^
						**LaCl3** ***vs*** **column (fold change)**
Filamin-A (fragment)	P21333	280739	74	16	17^b^ + 147^c^	0.02
						(4.0)
Coronin-1A	P31146	51678	171	40	2^b^ + 9^c^	0.02
						(15.0)
Pyruvate kinase isozymes M1/M2 (fragment)	P14618	57937	92	21	5^b^ + 28^c^	0.02
						(3950)
Ficolin-1	O00602	35078	78	17	2^b^	0.02
						(1.92)

^a^)Mann Whitney U-test. ^b^)Phosphorylation sites described in UniProtKB (http://www.uniprot.org) and in ^c^)PhosphoSitePlus (http://www.Phosphosite.org).

**Figure 2:**
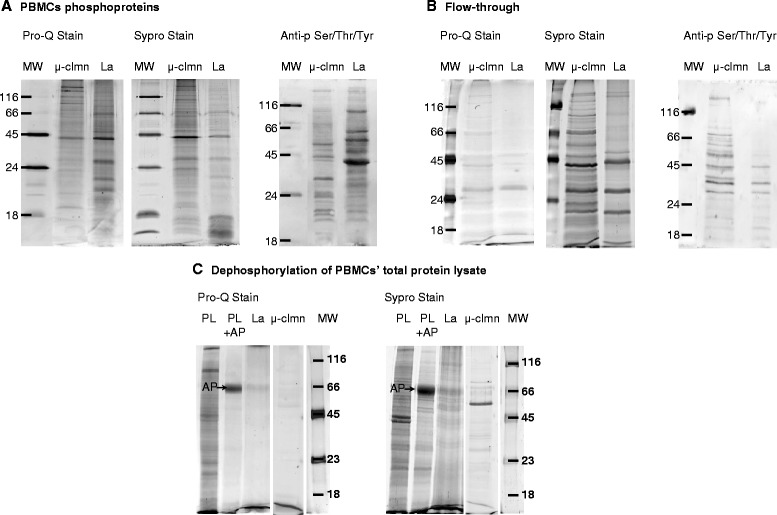
**Comparison between lanthanum and micro-column PBMCs’ phosphoprotein profiles. (A)** Sequential staining of PBMCs’ phosphoproteins enriched by LaCl_3_ (La) and micro-column (μ-clmn) and Western blot investigation of phosphoproteins using an anti-phospho-Ser/Thr/Tyr monoclonal antibody. Eluted proteins were separated by 1D-SDS-PAGE, and stained first with ProQ to visualize phosphoproteins, then with SYPRO to visualize total proteins. **(B)** The same analysis was performed on the corresponding flow-through. **(C)** Two identical aliquots of PBMCs protein lysate were both incubated with alkaline phosphatase (AP) in the same conditions. Then, the two aliquots were adjusted at pH = 7.4 (physiological pH) before being treated with lanthanum or micro-column. The entire amount of isolated proteins were separated by 1DE and stained first with ProQ, then with SYPRO. AP: alkaline phosphatase; PL: PBMCs protein lysate; PL + AP: dephosphorylated PL. MW markers: PeppermintStick™ Phosphoprotein MW Standards.

To further compare the two enrichment methods and to assess their specificity for phosphorylated proteins, phosphoproteins isolated with either method were analyzed by 1D gel electrophoresis and sequentially stained with Pro-Q® Diamond dye, the gold standard for gel-based detection of phosphoproteins, and subsequently, the gels were counter stained with SYPRO Ruby dye, for the detection of all proteins, and acquired. The comparison of the two lanes stained with Pro-Q (i.e. lanthanum *vs* micro-column enrichment) showed marked differences in the band distribution in the medium-lower part of the gel, where the lanthanum profile seemed to guarantee a higher yield (Figure 2A), which likely resembled 2DE pattern differences (Figures 1 and 2A). On the other hand, the SYPRO-stained profile, which detected total proteins, was roughly superimposable, in terms of number and distribution of bands, to the Pro-Q stained profile (Figure 2A) for each of the two methods, suggesting a quite good level of phospho-specificity of both enrichment approaches. In addition, the two phosphoprotein patterns were analyzed by Western blot using an anti-phospho-Serine/Threonine/Tyrosine monoclonal antibody (Figure 2A) which revealed differences in the intensity rather than in the number of detected bands (almost 60% match of bands which showed different intensities) between the two enrichment methods, resembling the findings of 2DE comparative analysis, in spite of the technical differences between the two analytical strategies (2DE and Western blot).

To assess the validity of the two enrichment strategies for PBMCs phosphoproteome analysis we wondered whether the supernatant of the precipitation was really depleted in phosphoproteins. Sequential staining with ProQ and Sypro dyes of the supernatants (flow-through) derived from lanthanum or micro-column treatment of two identical aliquots of the same protein lysate, pointed out the inability of both strategies to completely deplete phosphoproteins of the PBMCs’ protein sample (Figure 2B), although suggesting a better lanthanum ions capability.

**Scheme 1:**
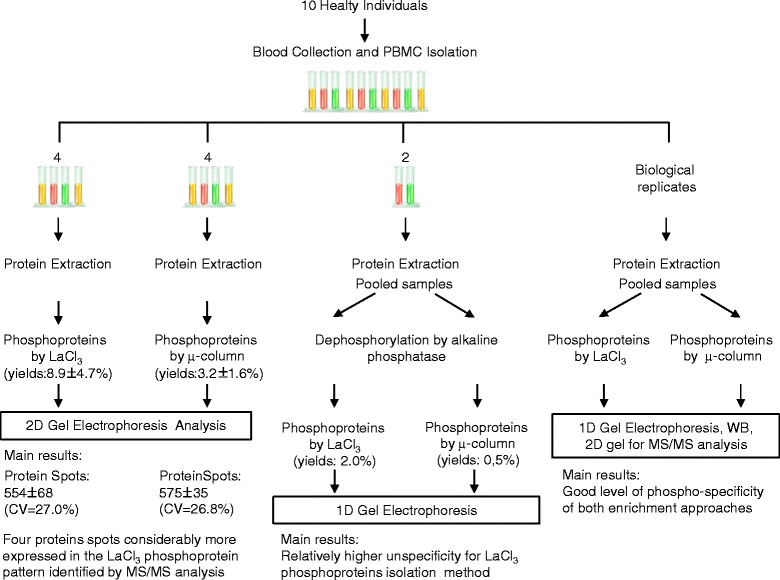
**Study design and main results.** μ-column: affinity micro-column.

Specificity of both enrichment methods was further investigated with dephosphorylated PBMCs protein samples. Two identical aliquots of a pool of PBMCs’proteins were incubated with alkaline phosphatase, the phosphatase with the broadest substrate specificity [15]. After dephosphorylation, the two aliquots were adjusted to pH7.4 (which was the pH of PBMCs’ protein lysate) and immediately treated with lanthanum chloride and the micro-column, respectively, to isolate phosphoproteins. The entire isolated protein contents were separated on 1D-gel (Figure 2C) and sequentially stained with ProQ and Sypro dyes. From Sypro stained gel, both isolation strategies showed a few not-phosphorylated protein bands, which revealed some degree of unspecificity for both enrichment strategies (Figure 2C). The yield of proteins isolated from dephosphorylated samples was 0,5% for micro-column and 2% for lanthanum chloride, suggesting a relatively higher unspecificity for the latter enrichment method, as we expected. Scheme 1 depicted the overall picture of the study design and the collected results.

Multiple factors may account for the differences in the phosphoprotein micro-column pattern compared to lanthanum. Since the present study was not intended to explore the mechanisms underlying the different specificity of the two enrichment methods, and no detailed information on the micro-column procedure is provided by the manufacturer, we may just suppose that a combination of chemical (multiple phosphorylations; pI; basic group of aminoacids and other PTMs) and stereochemical features could differentially contribute to the phosphoproteins capture. For instance, we may suppose that the protein steric hindrance can influence the quality of the phosphoproteins bound by the phospho-active site immobilized to the resin-column more than the free-lanthanum ions. Then, we may suppose that the easy accessibility of the lanthanum ions (dissolved in the protein solution) compared to the phospho-active sites bound to the column and the dynamics of the phosphoprotein isolation procedures (protein sample and lanthanum ions mixture vortexing *versus* protein sample run through the column) can influence the total amount of isolated phosphoproteins, and therefore their relative yields. Therefore, the two strategies could be complementary for an in-depth PBMCs phosphoproteome analysis.

## Conclusions

In conclusion, an in-depth analysis of intracellular pathways of PBMCs would be greatly favored by a thorough identification of phosphoproteins, and their isoforms, in the the 250-10 kDa mass range. This implies the need for a standardization of 2DE maps of PBMCs phosphoproteins. The present data show that phosphoprotein isolation by lanthanum ions and by micro-column are both a suitable preliminary step for 2DE phosphoproteome analysis of PBMCs, which is here described for the first time. In our experience, phosphoprotein isolation by micro-column provides a poorer but less unspecific enrichment strategy compared to lanthanum ions. On the other hand, lanthanum ions, besides to the relative higher unspecificity compared to the micro-column, presents the advantages of higher phosphoproteins yields, lack of limitations in the amount of protein loading, cheapness, ease of use, and rapidity of the entire enrichment procedure.

## Methods

### Materials

Acetonitrile (ACN), acetone, trifluoroacetic acid (TFA), trichloroacetic acid (TCA), DL-dithiothreitol (DTT), iodoacetamide (IAA), glycine, EDTA, Tris, endonuclease, phosphoprotease and protease inhibitors, lanthanum chloride, potassium dihydrogen phosphate, Coomassie Blue G-250, imidazole, alkaline phosphatase were purchased from Sigma (Sigma Aldrich St.Louis, MO, USA); urea, CHAPS, SDS, glycerol, acrylamide, ampholine, and Ficoll-Paque™ were purchased from GE healthcare (Uppsala, Sweden); Agarose, Pro-Q® Diamond Phosphoprotein Enrichment kit, Pro-Q® Diamond dye, SYPRO® Ruby and PeppermintStick™ Phosphoprotein Molecular Weight Standards were from Invitrogen™ (Carlsbad, CA); piperazine di-acrylamide (PDA), TEMED, Bio Rad Protein Assay, IPG strips, were from Bio-Rad Laboratories (Hercules, CA). Trypsin (sequencing grade modified) was from Promega (Madison, Wisconsin, USA). All solvents used were Ultra-Resi-Analyzed grade.

### Peripheral blood mononuclear cells isolation

PBMCs were isolated from whole blood (29,7 ± 2,3 mL) collected in lithium heparin from ten healthy individuals. The volunteers were ascertained as healthy by a general medical examination and were not taking drugs. The study was approved by the local ethical committee and an informed consent was collected from each voluntary blood donor. Isolation of PBMCs was performed within 2 hours after blood was drawn from healthy donors. Fresh blood was diluted with an equal volume of PBS pH 7.4/1 mM EDTA and PBMCs were isolated by density separation over a Ficoll-Paque gradient (460xg for 30 min). PBMC were then washed once with PBS, and twice with physiological saline solution (0.9% NaCl). Cells were then counted and their viability was assessed by trypan blue exclusion (>90% PBMC were viable). PBMCs numbers were obtained from each donation in a mean of 41 ± 13 × 10^6^ cells. All procedure involving cells isolation were conducted under sterile conditions. Total proteins were extracted by adding 500 μL RIPA buffer with 10 μL endonucleases, and 5 μL phosphoprotease and protease inhibitors to PBMC pellet. After the pellet was incubated on ice for 30 minutes, the cell lysate was centrifuged at 10,000xg at 4°C for 20 minutes. Protein concentration was assayed by Bradford method and proteins were stored at -80°C until use. Eight of ten PBMCs protein samples were singularly used for 2DE analysis, and all ten PBMCs protein samples were pooled for 1D gel electrophoresis analyses. All experiments were run in triplicate unless otherwise noted.

### Isolation of phosphoproteins

In the first approach, the procedure described by Pink and coworkers [6] was adapted to enrich the phosphoproteome of 4 randomly chosen PBMCs samples. Briefly, 1 M lanthanum chloride and 2 M potassium dihydrogen phosphate were added to the solution of total cellular proteins (0.5 - 2 mg) to precipitate phosphoproteins as described [6]. After the washing steps, the phosphoproteins were eluted from the pellet using a mixture of 25% 4 M Imidazole and 75% sample buffer (8 M urea, 2 M thiourea, 2% CHAPS, 1% DTT in water) and purified by cold acetone/20% TCA in water solution. Phosphoproteins were finally resuspended in IEF buffer [8 M urea, 2% w/v CHAPS, 0.5% ampholine (pH 3-10), 18 mM DTT, 0.002% w/v bromophenol blue (BBP)].

In the second approach, phosphoproteins of 4 PBMCs samples, different from those used for lanthanum ions precipitation, were enriched by Pro-Q® Diamond Phosphoprotein Enrichment kit of Invitrogen. Phosphoproteins were isolated according to the manufacturer’s protocol for 0.5-1 mg of protein extract. All samples were run at least in duplicate.

### 2D gel electrophoresis

Phosphoproteins isolated with the two different methods were then analyzed by 2D-PAGE. Briefly, IEF was carried out using immobilized 7 cm pH 3-10 gradient IPG strips, previously rehydrated with 125 μL IEF buffer overnight at 20°C. Fourteen or 200 μg phosphoproteins were loaded onto rehydrated IPG strips, for analytical or preparative gels respectively, and IEF was performed at 22 kV · h total produced by overnight run. After strips equilibration [16], the second dimension run was carried out using homemade polyacrylamide/PDA (12,5% T/2,6% C) gel on a Mini-ProteanTetraCell system (BioRad) as described [16]. Analytical 2-DE gels (at least two replicates for each sample) were stained with SYPRO® Ruby according to the manufacturer’s protocol and acquired with a PROXPRESS 2D scanner (Perkin Elmer Life Sciences, Cambridge, UK). Preparative 2-DE gels were stained with colloidal Coomassie Blue (0.02% CBB G250), and Image Master Platinum 2D software was used for image analysis of analytical 2D gels as previously described [16]. The fold change of protein expression between the two classes (LaCl_3_*versus* affinity micro-column) was calculated considering the mean of spot intensity (measured as the relative volumes of spots) of the 4 reference gels in each class.

### 1D gel electrophoresis

Two identical aliquots (0.5 mg) of the same pooled protein lysate were treated with lanthanum chloride and micro-column, respectively, to isolate phosphoproteins. Phosphoproteins isolated with either method, and the corresponding flow-through derived from phosphoproteins precipitation, were analyzed by 1D SDS-PAGE using homemade polyacrylamide/PDA (12,5% T/2,6% C) gel as previously described [16] and loading 10 μg sample in each lane. The proteins were stained following the manufacturer’s protocol for Pro-Q Diamond dye and gels were scanned with a PROXPRESS 2D scanner. Subsequently, after a rapid water washing step, the gels were counter stained directly with SYPRO Ruby dye and acquired.

### Western blot

Ten μg of phosphoproteins isolated with either method from pooled protein lysate, and the corresponding flow-through derived from phosphoproteins precipitation, were analyzed by Western blot (WB) using an anti-phospho-Serine/Threonine/Tyrosine monoclonal antibodies (Abcam, UK). Briefly, phosphoproteins were separated by 1D gel electrophoresis as previously described and blotted onto a nitrocellulose membrane. After the transfer (100 V, 1 h) the membrane was blocked in 5% BSA (PBS-T 0.1%) for 1 h at room temperature and then incubated overnight with primary anti-phospho-Ser/Thr/Tyr at 4°C. Signal band development and densitometric analysis were performed as described [16].

### Dephosphorylation of PBMCs proteins

Two identical aliquots of a pool of PBMCs’protein lysate (500 ug) were obtained by adding 500 μL RIPA buffer with 10 μL endonucleases, and 5 μL of the only protease inhibitors to PBMC pellet from two healthy individuals. Protein lysates were incubated with 700 U of alkaline phosphatase at pH 9, at 37°C for 16 hours. After that, the two aliquots were adjusted to pH7.4 with 0.6 N HCl and immediately treated with lanthanum chloride and the micro-column, respectively, as above described in the *Isolation of phosphoproteins* paragraph. The entire amount of isolated protein, were assayed by Bradford method, separated on 1D-gel and sequentially stained with ProQ and Sypro dyes, as above described in the *1D gel electrophoresis* paragraph.

### MALDI-TOF MS/MS analysis

After trypsin digestion [16], the peptide mixture of the selected protein spots was loaded onto the pre-spotted anchor chip (PAC, Bruker Daltonics, Bremen, Germany) and mass spectra were acquired on Autoflex III™ TOF/TOF200 instrument (Bruker Daltonics) as previously described [17]. Protein identification was achieved by database search via Biotools 3.2 and MASCOT search algorithm (http://www.matrixscience.com) against the MSDB, NCBInr and Swissprot databases using the following parameters: Homo Sapiens as taxonomic category, trypsin as enzyme, carbamidomethyl as fixed modification for cysteine residues, oxidation of methionine as variable modification, and one missing cleavage and 100 ppm as mass tolerance for the monoisotopic peptide masses and 0.3 Da mass tolerance for MS/MS analysis. Protein identifications were considered to be confident when the protein score of the hit exceeded the threshold significance score of 56 for PMF (*p* <0.05) and 27 (*p* <0.05) for MS/MS data, first “hit” protein was selected. The tryptic peptide mixture was further analyzed using a matrix combination of 2,6-dihydroxyacetophenone (DHAP)/diammonium hydrogen citrate (DAHC) (1/30) [18], loaded on MTP384 ground steel target (Bruker Daltonics) and acquired (Additional file 1). Phosphosite (http://www.phosphosite.org) was used to recognize the known phosphorylation sites, while NetPhos 2.0 Server was used to predict the phosphorylation sites of the identified proteins reported in Table 1.

### Statistical analysis

The results of the quantitative variables were expressed as mean ± SD. Differences between quantitative variables were tested by the Mann–Whitney U-test. P-values 0.05 were considered statistically significant. The Statview software package, SAS (5.0 version) was used for all analyses.

## Additional file

Additional file 1:
**Protein Identification by MALDI-TOF-MS/MS analysis.**

## Abbreviations

DHAP: 2,6-dihydroxyacetophenone
DAHC: Diammonium hydrogen citrate
MW: Molecular weight
