# Supplementary material for: Two dimensional gel phosphoproteome of peripheral blood mononuclear cells: comparison between two enrichment methods
Source: Proteome Sci. 2014 Sep 9;12:46. doi: 10.1186/s12953-014-0046-1 (PMC4177430; doi:10.1186/s12953-014-0046-1)
Supplement: Additional file 1: — Protein Identification by MALDI-TOF-MS/MS analysis. [file 12953_2014_46_MOESM1_ESM.docx]

**Additional file**

**Protein Identification by MALDI-TOF-MS/MS analysis**

Four protein spots differentially expressed between the two methods, and stained by ProQ® Diamond, were identified by MALDI-TOF-MS/MS as FLNA, CORO1A, KYPM and FCN-1(additional figure 1). The 2DE gels, loaded with 14ug of phosphoproteins enriched by lanthanum chloride, were first stained with ProQ (sensitivity of about 1-16 ng, depending on the phosphorylation state of the protein) and, after a rapid water washing step, were counter stained with SYPRO® Ruby (sensitivity about 0.25-1 ng) following the manufacturer’s protocols. Gels were scanned with a PROXPRESS 2D.

**
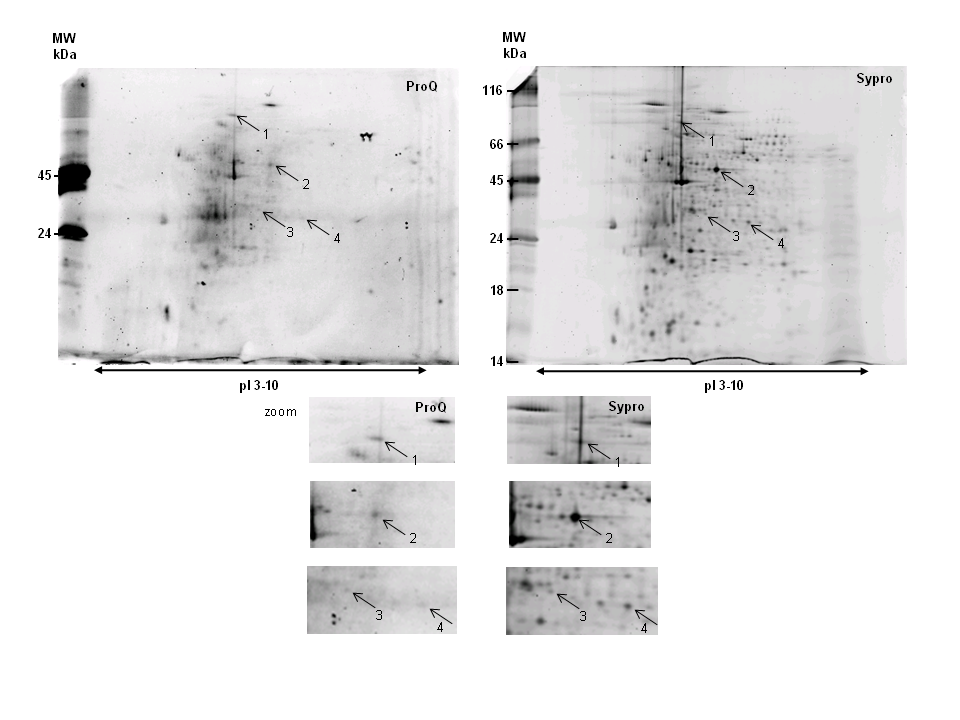
Additional figure 1.** 2DE combined with sequential staining. MW markers: PeppermintStick™ Phosphoprotein MW Standards. Arrows mark spots that are visible in both, the ProQ® Diamond and SYPRO® Ruby stained gel

To enhance phosphopeptide analysis the tryptic peptide mixtures were further analyzed using a matrix combination of 2,6-dihydroxyacetophenone (DHAP)/diammonium hydrogen citrate (DAHC) (Sigma Aldrich) (1/30) prepared as described [Hou J, Xie Z., Xue P, Cui Z, et al. Enhanced MALDI-TOF MS analysis of phosphopeptides using an optimized DHAP/DAHC matrix. J Biomed Biotechnol 2010, doi:10.1155/2010/759690 ]. The sequencing of at least 1 phosphopeptide for each protein identified has been reported. The CID MALDI-TOF-MSMS analysis of the four peptide mixture (corresponding to spot 1-4 in additional figure 1) identified the 1734 m/z as the Filamin A peptide (aa2150-2165) phosphorylated on S_2152_ (100% probability), the 855.4 m/z as the CORO1A peptide (aa. 394-400) phosphosphorylated on Y_396_, the 1277 m/z of KYPM (aa. 142-151) phosphorylated on Y_148_ and the 1169 m/z (aa.132-170) and the 1341 m/z (aa.131-170) as FCN1 peptides phosphorylated on Y_169_, as shown below. Positive ion MALDI mass spectra were acquired with AutoflexIII smartbeam in the reflectron mode. CID MS/MS operation parameters were: argon as gas; ion source 1, 6.02 kV; ion source 2, 5.32 kV; lens 3.02 kV; reflectron 1, 27.07 kV; reflectron 2, 11.52 kV; LIFT 1, 19.08 kV; LIFT 2, 4.71 kV. MS/MS protein identification was achieved by database search via Biotools 3.2 and MASCOT search algorithm (http://www.matrix.science.com) against the MSDB, NCBInr and Swissprot databases using the following parameters: Homo Sapiens as taxonomic category, trypsin as enzyme, carbamidomethyl as fixed modification for cysteine residues, oxidation of methionine, phosphorylation of Serine/Threonine or phosphorylation of Tyrosine as variable modification, and one missing cleavage and 50-100 ppm as mass tolerance for the monoisotopic peptide masses and 1.0 Da mass tolerance for MS/MS analysis. MASCOT automatic error tolerant search for MS/MS data of the expected phospho-peptides on the candidate protein target has been employed using combined PMF and MS/MS datasets. Mascot search results have been reported below as url format.

| **Supplemental TABLE 1 –** Proteins spot detected in PBMCs 2DE phosphoproteome identified by MALDI-TOF-MS/MS | | | | | |
| --- | --- | --- | --- | --- | --- |
| **GENE NAMES** | **Protein name** | **MASCOT score** | **Sequence Coverage (%)** | **No. of matched peptides (PMF)** | **Peptide sequenced (MS/MS)** |
| FLNA  (spot 1) | Filamin A | 74 | 16 | 33 | R.AEAGVPAEFSIWTR.E  K.FNEEHIPDSPFVVPVASPSGDAR.R  R.APSVANVGSHCDLSLK.I (**p**S_3_) |
| CORO1A  (spot 2) | Coronin-1A | 171 | 40 | 20 | R.DGGLICTSCR.D K.ADQCYEDVR.V  K.DRPHEGTRPVR.A  K.DG**p**YVPPK.S |
| KPYM  (spot 3) | Pyruvate kinase isozymes M1/M2 (up fragment) | 92 | 21 | 12 | K.IENHEGVR.R  R.LDIDSPPITAR.N  R.NTGIICTIGPASR.S  K.ITLDNA**p**YMEK.C |
| FCN1  (spot 4) | Ficolin-1 | 78 | 17 | 7 | R.MDGSVDFYR.D  R.RMDGSVDFYR.D  R.VDLVDFEGNHQFAK.Y  R.MDGSVDF**p**YR.D  R.RMDGSVDF**p**YR.D |

PC connected on internet: Double Click on the corresponding icone **“Mascot Search Results …” or “Peptide Summary Report…” or to visualize MS and MS/MS data:**

**FLNA,** Filamin A (fragment)

PMF

MS/MS SEQUENCING of 1533 m/z and 2467 m/z

MS/MS phosphopeptide sequencing of (pS) 1734 m/z

**CORO1A,** Coronin-1A

PMF

MS/MS SEQUENCING of 1138 m/z, 1155 m/z, 1319 m/z

MS/MS phosphopeptide sequencing of (pY)855 m/z

**KPYM,** Pyruvate kinase isozymes M1/M2 (up fragment)

PMF

MS/MS SEQUENCING of 953 m/z, 1197 m/z, 1359 m/z

MS/MS phosphopeptide sequencing of 1277 m/z

**FCN1,** Ficolin-1

PMF

MS/MS SEQUENCING of 1089 m/z, 1261 m/z, 1618 m/z

MS/MS phosphopeptide sequencing of 1169 m/z and 1341 m/z
